# Holographic sol–gel monoliths: optical properties and application for humidity sensing

**DOI:** 10.1098/rsos.172465

**Published:** 2018-05-02

**Authors:** Daniil A. Ilatovskii, Valentin Milichko, Alexander V. Vinogradov, Vladimir V. Vinogradov

**Affiliations:** SCAMT Laboratory, ITMO University, St. Petersburg, 197101, Russian Federation

**Keywords:** sol–gel chemistry, soft lithography, optical humidity sensor

## Abstract

Sol–gel monoliths based on SiO_2_, TiO_2_ and ZrO_2_ with holographic colourful diffraction on their surfaces were obtained via a sol–gel synthesis and soft lithography combined method. The production was carried out without any additional equipment at near room temperature and atmospheric pressure. The accurately replicated wavy structure with nanoscale size of material particles yields holographic effect and its visibility strongly depends on refractive index (RI) of materials. Addition of multi-walled carbon nanotubes (MWCNTs) in systems increases their RI and lends absorbing properties due to extremely high light absorption constant. Further prospective and intriguing applications based on the most successful samples, MWCNTs-doped titania, were investigated as reversible optical humidity sensor. Owing to such property as reversible resuspension of TiO_2_ nanoparticles while interacting with water, it was proved that holographic xerogels can repeatedly act as humidity sensors. Materials which can be applied as humidity sensors in dependence on holographic response were discovered for the first time.

## 1. Introduction

Nowadays, materials with structured periodic micron-scale surfaces or superstructures are widely used both in industry and science. For instance, these are applied as holographic materials [1,2], distributed feedback resonators [3], diffraction gratings [4], guides of stem cells growth [5] and others. Particularly, these structured materials can be prepared by the soft lithography route, which was chosen in this study because of simplicity of its technology, absence of additional equipment and low cost of production. This method is commonly used for structuring polymers [6] or organo–inorganic material [7,8] surfaces. These materials are known to have low refraction, hence one is faced with unsatisfying visibility of diffraction. Although it is possible to increase the refractive index (RI) of polymers [9], the cost of the final products increases drastically. On the other hand, inorganic high-refractive sol–gel systems based on high electron density oxides could provide bright visible effect. Moreover, impregnating super black materials such as multi-walled carbon nanotubes (MWCNTs) [10] within a sol–gel matrix can strongly increase the diffraction colour by increasing total RI and lending absorption properties.

The soft lithography route has several types [6], but in this work a modified method based on replica moulding was developed, because methods for the production of inorganic monoliths with superstructure on their surfaces under normal conditions (atmospheric pressure/room temperature) are unknown. Previous investigations [11,12] aimed at obtaining thin structured films and included a calcining stage, which makes the production more expensive.

Furthermore, soft lithography was used for the production of superhydrophobic surfaces from sol–gels [13]. In that work, the replication of structure was not accurate, which was enough for the repulsion of water, but unsuitable for optical materials considered in this work. Optical materials were also created, for instance, for microlens array production [14], where replication took place with high fidelity. However, UV curing and heat treatment were employed there in order to stabilize the structure. On the other hand, when mesoporous sol–gel films were used for waveguide patterning [15,16], low RI was their advantage, in contrast to the present study where xerogels with high refraction are needed. To make things clear, soft lithography includes the fabrication of elastomeric pattern—master [17]. As it is rather inconvenient, this stage was also avoided, because polyethylene terephthalate (PET) holographic films replaced special stamps.

SiO_2_ was chosen in this study as an appropriate sol–gel system, because it is possible to synthesize it quickly and simply, and its xerogels are transparent and relatively massive [18]. However, RI of silica is known to be rather low [19], hence it was necessary to find some highly refractive non-silica materials. According to the review [20], TiO_2_ and ZrO_2_ are suitable high electron density oxides for the production of transparent monoliths.

Dried solid monoliths of silica, titania and zirconia sol–gels are known to crack with structural collapse [21–23]. To fix it, some methods include addition of organic stabilizers to matrices [11,22,24,25], but in this study additives are to be abandoned, because they decrease transparency or RI. Consequently, the problem of cracking is to be solved further. The most relevant methods of synthesis are based on condensation of very small nanoparticles, which are peptized by acids, because no organics are used in the process, systems are transparent after condensation and gelate physically.

We show here—using optical and structural measurements—that sol–gel matrices with high RI can be applied for soft lithography of holographic colourful diffraction at atmospheric conditions and also that after entrapment within MWCNTs, they quite significantly enhance the visibility of the diffraction colour, making it much brighter. SiO_2_, ZrO_2_ and TiO_2_ were used for obtaining structured holographic monoliths, and PET holographic films were taken as a source of ordered channels to be deposited for visible diffraction effects.

Given high visibility of the holographic effect for prepared monoliths, which can be easily detected by naked eye, we suggest testing them as reversible humidity sensors. Humidity is the basic parameter characterizing environmental conditions. It is often important to know whether level of humidity was exceeded during storage, shipping and production of some casual products, for instance, drugs, clothes, shoes, food, electronics and hygroscopic substances, e.g. fuels and explosives. Visual sensors of humidity, such as those based on metal organic frameworks [26], photopolymer holograms [27] and superparamagnetic photonic colloids [28] are well known, but their production is always a complex multistage process. Recently, a diffractive optical sensor was developed employing sol–gel methodology for detection of both H_2_O and NH_3_ in the environment [29]. However, the nature of its phenomenon is based on different processes. In the idea we propose in this work, qualitative detection of air humidity is achieved by change of effective refractive index (RI) because of water absorption and change in diffraction grating's geometry along all three linear dimensions. This change could influence diffraction efficiency and its visibility by naked eye, respectively. In addition, relaxation of the proposed phenomenon is almost absent without drying, so our samples potentially could save prehistory of conditions which they were placed in. Diffractive sensors from [29] are composite materials: NiCl_2_ and CuCl_2_ nanoparticles dispersed in SiO_2_ sol–gel matrix. Holographic efficiency varies there because of appearance of clusters, which change effective RI and morphology of surface [30]. The relaxation here is fast enough to provide real-time monitoring of NH_3_ level in the atmosphere. Diffraction grating is etched on surface of samples just to enhance this effect by rise of active area, which improves the sensitivity of sensors. Also, systems without Ni and Cu salts do not show sensor properties, as well as sensors which were prepared in this study, and cannot be used in this way, because of difference of interaction mechanisms.

Some qualitative colourful sensors were also produced with outstanding visibility, brightness and simplicity of production [31]. In spite of their advantages, reversibility of humidity indication requires high temperature treatment, so their applicability is limited. Next, detailed study of recent publications about holographic sensors, especially made of photopolymers [32,33], helped authors to define that this field faces the problem of irreversibility even after short treatment by relative humidity 70%.

In addition, mentioned sensors are targeted in real-time humidity estimation. On the contrary, sometimes it is more important to know prehistory of environmental conditions, which shows the presence of environmental violations in the past. Here we will demonstrate the possibility of application of our materials for this purpose.

## 2. Experimental details

### 2.1. Material and methods

Tetramethylorthosilicate (TMOS) (98%, Aldrich), titanium tetraisopropoxide (TTIP) (97%, Aldrich), zirconium (IV) propoxide solution (70%, Aldrich), benzyl alcohol non-aqueous (99%, Chemmed), acetic acid (100%, Chemmed), nitric acid (65%, ACROS Organics), hydrochloric acid (36%, Chemmed), propanol-2 (Chemmed), diethyl ether (Chemmed), ethanol, deionized water (less than 5 µS cm^−1^), MWCNTs (Dealtom; outer diameter is 49.3 ± 0.45 nm and 72.0 ± 0.45 nm; internal diameter is 13.3 ± 0.45 nm; upper bound of length is 5 µm; specific surface area is 97.55 ± 0.02 m^2 ^g^−1^), holographic PET film.

### 2.2. Sol–gel synthesis

#### 2.2.1. Silica

Sol–gel was produced by hydrolysis and polycondensation of tetramethylorthosilicate. According to [34], an acidic route was chosen with TMOS as a precursor. Ethanol as a diluent was replaced by isopropanol for decelerating its further evacuation from the system, so crack emergence could be avoided. The ratio of reagents was as follows: TMOS 1 ml, isopropanol 1 ml, water 1.6 ml, HCl (36%) 0.032 ml. First, TMOS was diluted in propanol-2. Next, the dilution was added to water, which contained hydrochloric acid, under stirring for 5 min at room temperature. Finally, a transparent SiO_2_ sol was obtained.

#### 2.2.2. Titania

TiO_2_ sol was synthesized by a modified sol–gel method [35]. TTIP (3.73 ml) was diluted in 2.8 ml of propanol-2. Then, the solution was added dropwise to deionized water (23.31 ml), which contained nitric acid (0.163 ml). The synthesis was carried out at 80°C under continuous stirring (1000 r.p.m.) for 1 h. Milky suspension, which was obtained after this procedure, turned into a stable white translucent sol after 72 h upon being stirred vigorously at room temperature.

#### 2.2.3. Zirconia

According to a procedure originally used in [36], zirconia nanoparticles were produced in nitrogen atmosphere (inside a glove-box) by non-aqueous technology. Precursor (zirconium (IV) propoxide solution) in an amount of 2.4 mmol was dissolved in benzyl alcohol (BnOH) (18.9 mml) in a Teflon beaker. After that, it was placed inside a steel autoclave and heated to 220°C in the furnace. Thermal treatment was applied for 72 h. Next, diethyl ether was poured into the liquor involved, until a white precipitate was formed, which was separated from the liquid by centrifugation for 10 min at 10 000 r.p.m. Then, the precipitate was washed in ethyl alcohol twice. Finally, the sediment was peptized in acetic acid (approximately 2 × 10^−3^ mol per 10 ml of acid) under continuous stirring for 24 h.

### 2.3. Multi-walled carbon nanotubes@sol–gels

For the preparation of samples, which contain MWCNTs, the latter were sonicated in as-made sol–gel systems for 2 h at 60°C and were evenly distributed over their volume in an amount of 3 wt%. Long ultrasonic treatment of MWCNTs in sols resulted in their partial oxidation and hydrophilization [37], so stable black water dispersions were finally obtained.

### 2.4. Preparation of holographic sol–gel monoliths

**Figure 1.**
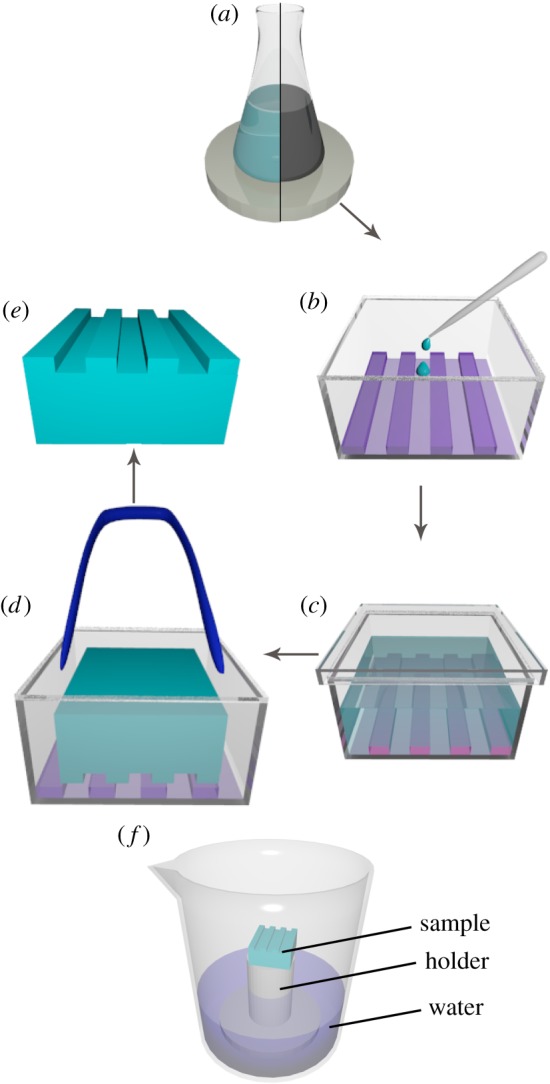
(*a–e*) Process diagram of the production of holographic monoliths: (*a*) heating the sol with (right) or without (left) MWCNTs, (*b*) filling the form with holographic film at its bottom by viscous gel, (*c*) drying of the gel for 72 h, (*d*) taking the dried monolith out of the form, (*e*) obtained xerogel monolith; (*f*) system for humidity sensing.

It is important to note that the production of holographic monoliths was carried out at atmospheric temperature and pressure without using a heating stage. Figure 1*a–e* shows the scheme of the soft lithography route. At first, the prepared sol–gel system (SiO_2_, TiO_2_ or ZrO_2_) or system with dispersed MWCNTs was poured into a conic flask in an amount of 5 ml and heated to 60°C under vigorous stirring, so that extra solvent was evaporated and gelation process was intensified (figure 1*a*). After 40 min, when nearly 1.5 ml of viscous gel remained in the flask, it was poured into a special polypropylene form with master (PET holographic film) at its bottom, so that the surface of the future monolith replicated relief was in the negative phase (figure 1*b*). Schematically, the form shown is box-shaped, but experimentally it should be round, because if it had corners, tensions in these corners would have resulted in collapse of xerogel. Directly afterwards, the form was covered non-hermetically by a plastic cover, and monolith was dried for approximately 72 h in a closed container, hence this process occurred slowly and air instability was avoided, as shown in figure 1*c*.

As a result, xerogel with one structured surface was obtained. Since the size of monolith is smaller than that of the form, and adhesion between the gel and PP walls of the form is rather weak, the monolith is easily separated from the walls and holographic film (figure 1*d*). Finally, both pure and MWCNT-doped monoliths were produced (figure 1*e*).

Since sol–gel glasses are quite fragile, the most important thing for the production of crack-free monoliths is to evaporate solvent as fully as possible before depositing on a holographic film, because high concentration reduces shrinkage of the further monolith and minimizes the possibility of structural collapse. The rest of the solvent has to be taken out of system upright and very slowly, so that shrinkage basically occurs in the form of vertical compression, and holographic structure at the bottom of xerogel is preserved.

### 2.5. Test for humidity sensing

First, holographic xerogel was placed on a holder inside a beaker with water at its bottom, according to the scheme shown in figure 1*f*. Next, beaker was covered hermetically by parafilm and was put inside an oven at 50°C for 6 h. After that, the sample was taken out of oven and beaker and its surface was investigated by atomic force microscopy (AFM). Finally, the sample was dried in vacuum for 5 h to check the reversibility of its sensor properties and was tested by AFM again.

### 2.6. Laser diffraction test

**Figure 2.**
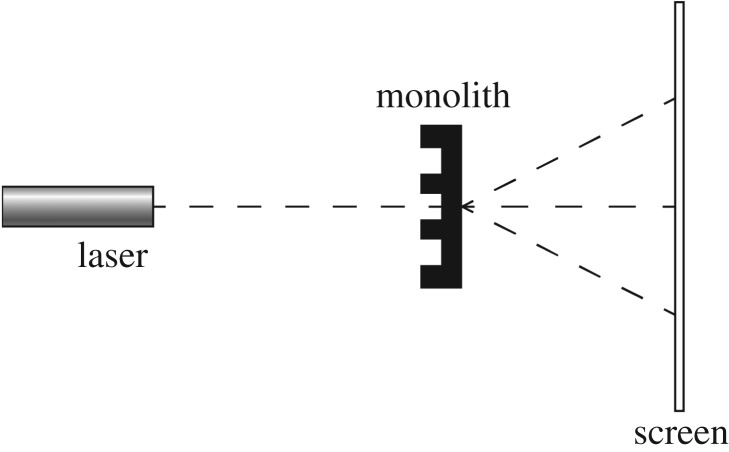
Humidity evaluation via laser diffraction test.

The test was carried out in a dark room to provide maximal visibility of laser beam diffraction by the holographic monolith. For this purpose, a planar monolith with a thickness of 0.5 mm was produced. Both green laser and holographic xerogel were fixed at the same level, as well as the holographic plane of material situated perpendicularly to the direction of laser beam, as shown in figure 2. The rays outgoing from the monolith were incident on the black screen, and the distance between the monolith and the screen was 5 cm.

In order to observe hygroscopic properties of the holographic monolith, it was placed in medium with 90% humidity, and photographs of the screen were taken at times 0, 30 and 60 min of treatment. Finally, the monolith was dried in vacuum and put in the installation again.

### 2.7. Characterization

Surface structures of obtained materials were investigated using a Tescan Vega 3 scanning electron microscope and a Solver Next atomic force microscope (by semicontact topography method using noncontact cantilever).

To provide RI measurements of all the sols, films were prepared from solutions by coating on microscope slides 26 × 76 mm (Paul Marienfeld, Germany) using a Meyer rod. After coating, the samples were dried at 60°C for 20 min to evaporate solvent and to make homogeneous xerogel films. Next, refraction measurements were made by the confocal optical scheme (more detailed information can be found in [8]).

## 3. Results and discussion

**Figure 3.**
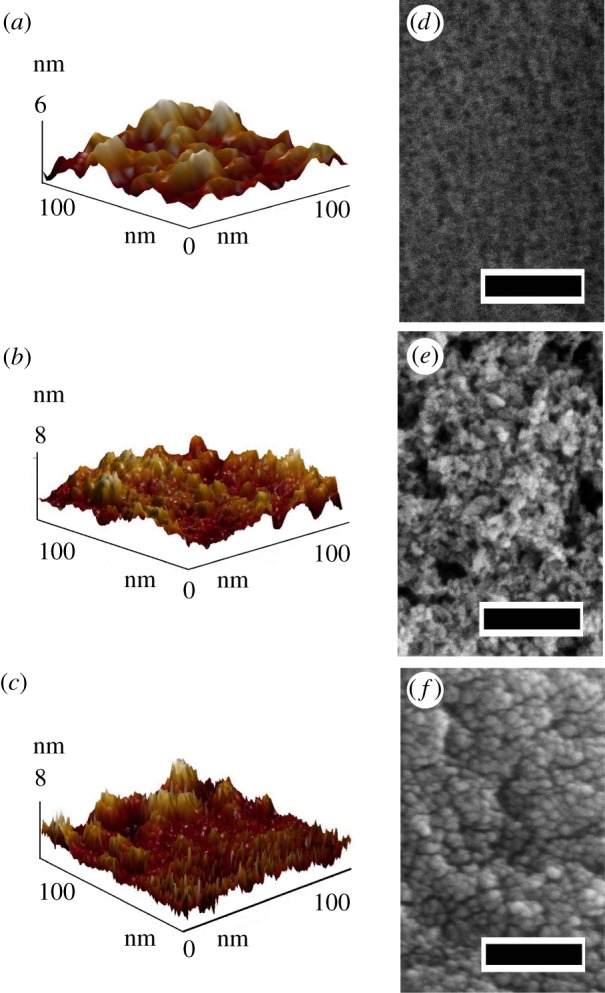
(*a–c*) AFM topography images of planar surfaces of silica, titania and zirconia, respectively; (*d–f*) SEM images of planar surfaces of silica, titania and zirconia, respectively (scale bar = 100 nm).

Before holographic monoliths were prepared, the ability of the synthesized sol–gel systems to form dense bulks, which consist of close-packed nanoparticles of respective oxides, was studied. As illustrated by the AFM data, non-structured films made of original materials have a flat homogeneous smooth morphology and consist of nanoscale particles near 10 nm (figure 3*a*–*c*). These particles pack close to each other due to slow condensation and form agglomerates with a linear size of a few cm. Despite rough appearance of the surfaces depicted in figure 3*a*–*c*, z-scale bar is of the order of nanometres, and their morphology is really plane and uniform. More detailed information on the size and morphology of nanoparticles is provided by scanning electron microscopy images (figure 3*d*–*f*). From the obtained data it is clear that the dense structure of xerogels is formed by particles with sizes of less than 10 nm. The combination of nanoparticle size and close packing minimizes the contribution of air intercalation to optical properties of materials, so that the RI of produced sol–gel systems becomes very close to the bulk materials.

After that, three issues were addressed at the following stage of the research. Do the monoliths, which were obtained at the first stage, replicate the structure of a holographic film, according to figure 1? Wherein does an optical property, such as colourful diffraction, of monoliths change for different materials? Finally, is it possible to enhance the brightness and visible holographic response, when corresponding dopants are added to the system?

**Figure 4.**
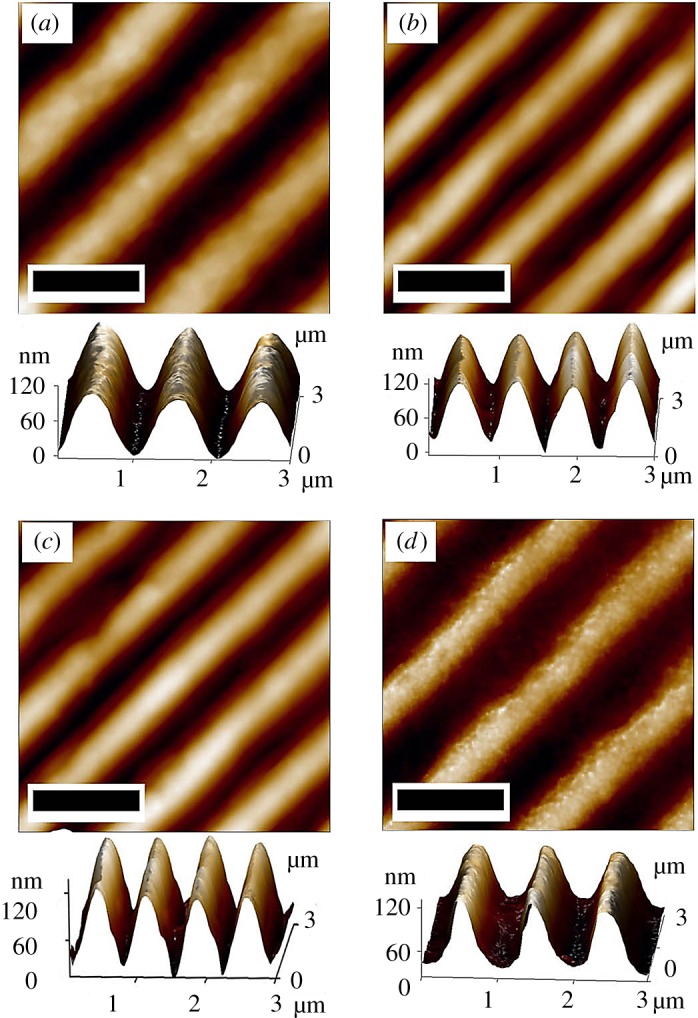
AFM topography image of the superstructures of: holographic film (*a*); replicated SiO_2_ (*b*), TiO_2_ (*c*) and ZrO_2_ (*d*) surfaces (scale bar = 1 µm).

The structure of holographic monoliths was investigated to describe the first issue. Figure 4 illustrates AFM analysis data of the original holographic film, which was used as master structure (figure 4*a*), and replicated silica, titania and zirconia xerogels (figure 4*b*–*d*, respectively). It can be seen that microscopically the superstructure of master consists of uniform wavy grooves with a 1 µm period. The height of channels is approximately 120 nm. The measurements of the topography of xerogels were taken after additional drying of monoliths in vacuum by desiccator with a Buchi V-300 pump (final vacuum 5 ± 2 mbar) for 24 h. This procedure took place in order to evacuate the remains of solvents from xerogels. The form of grooves is stated to be imprinted on the surface of monoliths very accurately, because their height is the same as that of the replica: approximately 120 nm. However, the width of channels declined roughly by 25% in silica and titania (figure 4*b*,*c*). Shrinkage of zirconia's channels is different, and their width declined less substantially (figure 4*d*). We assume that total evaporation of solvent (benzyl alcohol) from this system did not happen because of its high boiling temperature.

As it was not enough to vacuum xerogel for 24 h and the solvent remained in the system, shrinkage did not pass completely. This phenomenon can lead to a decrease in effective RI of a material, which will decrease the holographic visibility.

**Figure 5.**
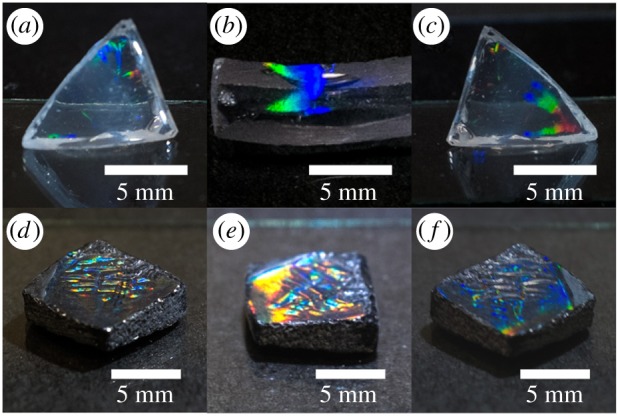
Photographs of produced monoliths: (*a*) SiO_2_; (*b*) TiO_2_; (*c*) ZrO_2_; (*d*) 3 wt% CNT@SiO_2_; (*e*) 3 wt% CNT@TiO_2_; (*f*) 3 wt% CNT@ZrO_2_.

Figure 5 shows the appearance of the obtained monoliths. Here, it can be clearly seen that xerogels can be formed mechanically from round to any desired shape, for instance, triangle (figure 5*a*,*c*), square (figure 5*d*–*f*) or parallelepipedal (figure 5*b*) with a linear size of around 1 cm. From the photographs it is obvious that the dimmest effect is seen for the SiO_2_ monolith; ZrO_2_ is intermediate and the maximum diffraction effect is observed on the titania surface. Because of aesthetic appearance of materials, it can be further applied in jewellery, replacing semiprecious stones. Moreover, it is assumed that due to this specific structure the products may act as photocatalysts [38], resonators [3] or sensors [39].

The next logical step was to search for some methods to gain the holographic optical response. To this end, we selected the addition of MWCNTs for two reasons: these increase RI of composite system [40] and lend absorbing properties to holographic grating, because MWCNTs are closer to blackbody model than any other materials, and unlike carbon black nanotubes can absorb light between 0.2 and 200 µm [41]. Topography of holographic monoliths doped by MWNCTs is not provided in the paper, because these do not change the morphology of holographic surfaces. Figure 5*d*–*f* shows that colourful diffraction visibility of MWNCT@SiO_2_ is higher than that for transparent undoped silica. The same situation is observed upon comparing MWNCT@TiO_2_ to pure TiO_2_ and MWNCT@ZrO_2_ to pure ZrO_2_.

**Figure 6.**
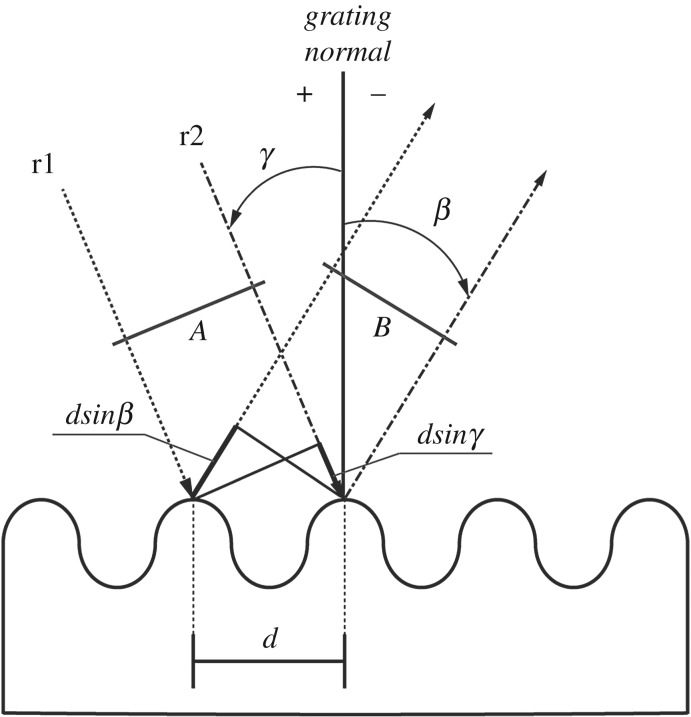
Diagram of diffraction for planar wavefronts. r1, r2 are the parallel rays; *d* is the grating period; A and B are the wavefronts of incident and diffracted light, respectively; *γ* is the angle of incidence of r2; *β* is the angle of diffraction of r2. Since *γ* and *β* are measured anti-clockwise from the grating normal, *γ* is greater than 0 and *β* less than 0.

Next, we will try to explain differences in holographic brightness among the synthesized systems. Since the channels illustrated in figure 4 act as a reflection diffraction grating, the colourful effect is visible (figure 5). According to Palmer & Loewen [4], geometrically diffraction is characterized by a diagram (figure 6). Two parallel rays r1 and r2 are incident on grating with distance *d* (equal to grating period). These rays are in phase with each other at wavefront A. The difference in paths of rays is *d*(sin *γ *+ sin *β*), because *γ* > 0 and *β* < 0. If the difference is equal to *mλ* (*m* is the diffraction order and *λ* is the wavelength), the light will be in phase at wavefront B, and constructive interference will take place. Otherwise, interference is destructive. Overall, the main equation of grating is:

3.1 mλ=d(sin⁡γ+sin⁡β).

One should use equation (3.1) for the systems with perpendicular incident light. Otherwise, Equation (3.2) is relevant:

3.2 mλ=dcos⁡ε(sin⁡γ+sin⁡β),

where *ε* is the angle between the direction of incident light and the plane perpendicular to the channels at the grating centre.

**Figure 7.**
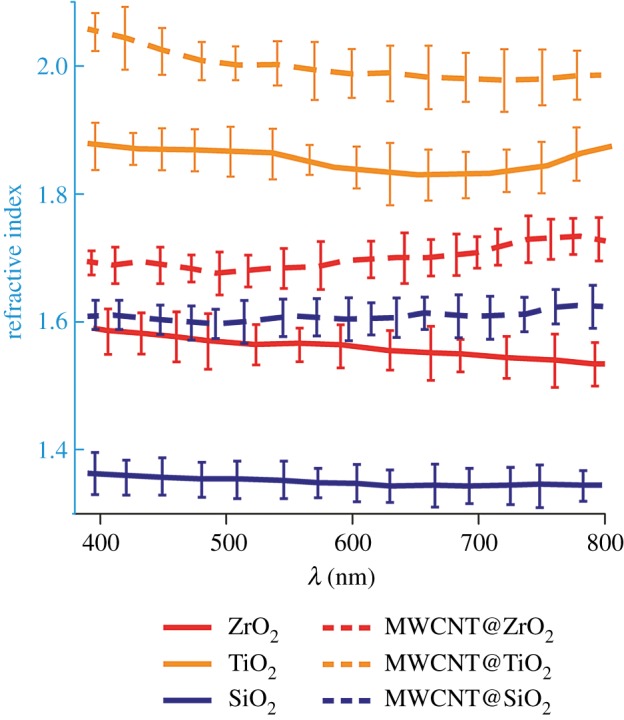
Refractive indices of pure and doped sol–gel systems of silica, titania and zirconia.

The holographic visible effect is characterized by hologram diffraction efficiency. According to its definition [4], holographic efficiency (*η*) of reflection grating rises if reflectance of material grows. Since reflectance is proportional to RI according to Fresnel equations [42], the material with the highest RI should have the highest holographic efficiency. RI measurements are shown in figure 7. The graph shows that RI of pure materials (solid lines) tends to increase in the order SiO_2_–ZrO_2_–TiO_2_, hence, *η*(SiO_2_) < *η*(ZrO_2_) < *η*(TiO_2_). RI of zirconium dioxide declined in comparison with previous investigations [43], which proves that system still contains extra solvent, benzyl alcohol, and it contributes to the total RI of xerogel.

The next trend shown by the graph is that RI of systems which were doped by nanotubes (dotted lines) grows noticeably, and despite the fact that absorption should decrease reflection properties, the visibility of holography rises, as seen in figure 5*d–f*, if compared with undoped samples, figure 5*a–c*, which means that *η*(MWCNT@sol–gel) > *η*(undoped sol–gel).

To summarize the previous paragraph, the brightest holographic effect is visible on the surface of titania with MWCNTs, so it was used for continuous qualitative tests of environmental humidity. We assumed that it can be applied in this way, because TiO_2_ xerogel is reversibly hygroscopic and resuspends by influence of water vapour. Thus, surface wavelike structure should expand, so that holographic response should weaken or totally disappear. On the other hand, if vapour treatment is not critical, the specific structure will not disappear from surface irreversibly, and water will evacuate from material after vacuum drying. This should return the visibility of holographic effect.

**Figure 8.**
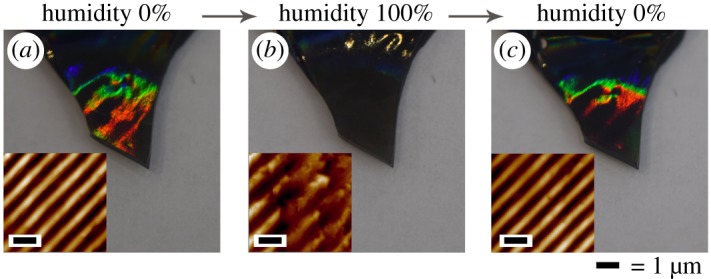
Digital photographs and AFM topography of: (*a*) original titania doped by MWCNTs monolith, (*b*) monolith after 6 h of humid treatment, (*c*) monolith after 24 h of drying (AFM scale bar is 1 µm).

Using AFM data and optical photographs, figure 8 illustrates how MWCNT@titania monolith acts as a reversible humidity sensor. The original monolith xerogel is shown in figure 8*a*, where bright colourful diffraction is seen because of homogeneous wave grooves of surface, which act as a diffraction grating. After the procedure of standing in humid environment, which is described previously by subsection ‘Test of humidity sensitivity', the holographic effect is poorly seen only at the top of sensor (figure 8*b*). According to its topography, partial degradation of grating happened, proving our assumption. Furthermore, channels of swollen monolith expanded, which explains again that ZrO_2_ in figure 4*d* did not desiccate fully because of similar expanded shape of grooves. As the sensor underwent significantly humid conditions, it absorbed water molecules from gas phase, which resulted in swelling of sample, so that geometry of diffraction grating has changed. Furthermore, absorbed water contributes to effective RI of the surface, so that it declines. However, after one day drying in desiccator under vacuum, the grating and visibility recovered again (figure 8*c*). This happened because swelling process is reversible (water exits from the material) and resuspension process does not take place after the above-mentioned treatment. Similar phenomenon was observed for previously developed sol–gel–sol biosensors based on alumina [44]. However, visible diffraction of sensors placed in aggressive humid environment for 10 or more hours degraded irreversibly, because the surface of material resuspends and diffraction grooves disappear. This fact can also be useful for demonstrating that humidity conditions during storage or some other process of any object were not met, if sensor was placed together with this object.

**Figure 9.**
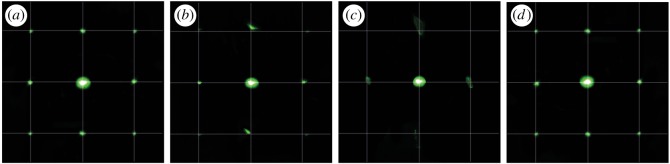
Picture of the screen from laser diffraction installation: (*a*) 0 min, (*b*) 30 min, (*c*) 60 min of 90% humidity treatment, (*d*) picture after vacuum drying for 5 h.

For more accurate information the test with laser beam diffraction was carried out. The picture from the screen is depicted in figure 9. Before humidity treatment (figure 9*a*), the picture of diffraction shows uniform deviation of laser points from the main direction of beam. The diffraction goes homogeneously in all directions, as shape, brightness and size of points are close to being equal both at vertical and horizontal deviation from main beam direction. The intensity of diagonal deviations is weaker, but diagonal points are equal too. This proves that the holographic surface is uniform and significant defects are absent. Figure 9*b* illustrates the picture after 30 min of 90% humidity treatment. Since the xerogel degraded during these conditions, points at horizontal and vertical directions are partially blurred and deviated, and intensity of points from diagonals decreased significantly. Figure 9*c* depicts the situation after 1 h of treatment. Here it is seen that dots of horizontal and vertical deviations became spots without clear shape. Moreover, diagonal dots disappeared totally, which shows high degradation of the holographic surface. After that, the sample was dried in vacuum for 5 h and put again in the installation from figure 2. The diffraction picture is depicted in figure 9*d*, where it is seen that the system is highly reversible and returned to its state at the beginning. The procedure of 1 h treatment in atmosphere with 90% relative humidity followed by 5 h drying was repeated 10 times. Degradation of surface and irreversible blurring of laser diffraction picture appeared only partially. Anyway, our sensors are expected to be applied one time and are not required to have highly reversible property.

Our monolith xerogels can separate the incident laser radiation, and the efficiency of this process strongly depends on amounts of water absorbed by the sample from air. Since this process is reversible, the humidity sensing tests can be provided nearly 10 times.

Thus, a material with dependence of humidity on holographic response is discovered for the first time, and the authors hope that this invention will diversify methods of fast humidity determination, as well as the direction of applying holographic materials.

## 4. Conclusion

Overall, the route of combining sol–gel synthesis and soft lithography was demonstrated for the production of transparent holographic silica, titania and zirconia monoliths. Holographic film was used as a template to avoid an additional stage of master obtainment. Owing to the method involved, it was possible to succeed in completely eliminating the power-consuming stage of calcination and to synthesize monoliths at atmospheric pressure. Addition of 3 wt% dispersed MWCNTs improved visibility of colourful diffraction. The brightest holographic effect is seen on the surface of TiO_2_ entrapped by carbon nanotubes because of the highest refraction of the material. This material is shown to act as a qualitative humidity sensor because of its holographic visibility, hygroscopicity and ability to resuspend reversibly.

Additionally, holographic xerogels can separate incident light, and this property can be applied for determining humidity, because the efficiency of separation depends on water absorption from air. A material with such property was produced in this study for the first time.

## Data accessibility

All the data are within the article and the package at Dryad: https://doi.org/10.5061/dryad.405hq [45].
