# Correction to ‘Holographic sol–gel monoliths: optical properties and application for humidity sensing’

**DOI:** 10.1098/rsos.181541

**Published:** 2018-10-10

**Authors:** Daniil A. Ilatovskii, Valentin Milichko, Alexander V. Vinogradov, Vladimir V. Vinogradov

*R. Soc. open sci.* **5**, 172465. (Published 2 May 2018). (doi:10.1098/rsos.172465)

Figure 5 in the published paper is incorrect. The correct figure is shown below:

**Figure 5.**
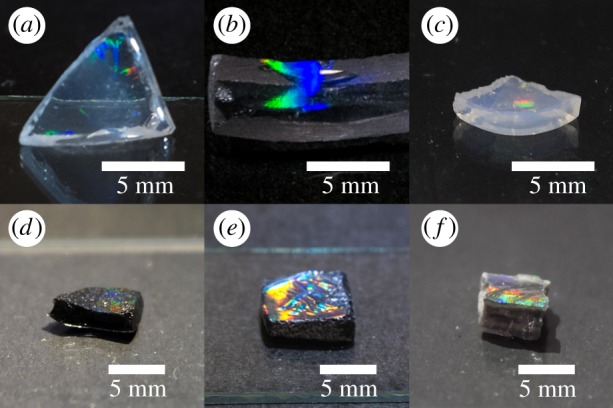
Photographs of produced monoliths: (*a*) SiO_2_, (*b*) TiO_2_, (*c*) ZrO_2_, (*d*) 3 wt% CNT@SiO_2_, (*e*) 3 wt% CNT@TiO_2_ and (*f*) 3 wt% CNT@ZrO_2_.

